# Evaluation of fractionated and repeated sonodynamic therapy by using dual frequency for murine model of breast adenocarcinoma

**DOI:** 10.1186/s40349-015-0031-x

**Published:** 2015-06-24

**Authors:** Mahboobeh Alamolhoda, Manijhe Mokhtari-Dizaji

**Affiliations:** Department of Medical Physics, Tarbiat Modares University, P.O. Box: 14115-133, Tehran, Iran

**Keywords:** Sonodynamic therapy, Dual frequency, Hematoporphyrin, Dose repetition, Dose fractionation, Breast adenocarcinoma

## Abstract

**Background:**

Sonodynamic therapy (SDT) is a new approach for cancer treatment. Repair by reoxygenation induces cell damage in all treatment which uses photo- and sonosensitizers. In this study, the in vivo antitumor effect of dual-frequency sonication is investigated at low-level intensity and hematoporphyrin (Hp). It is used for the treatment of spontaneous breast adenocarcinoma of Balb/c mice with a variety of dose repetition and fractionation regimes.

**Methods:**

Eighty tumor-bearing mice were divided into eight groups, the control group (A); the sham group (B); the injection of Hp alone group (C); 30-min dual-frequency sonication with Hp injection in one repetition at the first day group (D); two repetitions at the first and sixth days group (E); three repetitions at the first, sixth, and twelfth days group (F); four repetitions at the first, sixth, twelfth, and eighteenth days (30 min/repetition) group (G); and the fractional treatment group treated by dual-frequency sonication and Hp injection at the first, third, sixth, and ninth days (7.5 min/fraction) (*H*). For each group, the tumor growth delay was calculated during 30 days after treatment. These tumors were studied histopathologically.

**Results:**

The results show that the treatment with ultrasound dose repetition in two, three, and four times (E, F, and G groups) were effective in delaying tumor growth compared with one-time sonication (D group) (*p* < 0.05). Also, the ultrasound dose fractionation is more effective in decreasing the tumor growth rate compared with the ultrasound dose repetition in four repeats and in one repeat from the 12th to the 30th day (*p* < 0.05). Histopathological studies indicated that the mitotic activity of tumor cells was reduced following treatment with four fraction and four repetition protocols.

**Conclusion:**

The ultrasound dose fractionation and repetition technique with dual-frequency sonication can have a useful therapeutic effect in sonodynamic therapy with the possibility of use in future clinical applications.

## Introduction

Breast cancer is one of the most deadly diseases for women [1]. Over the years, three procedures for cancer therapy have mostly been surgery, radiation, and chemotherapy [2]. Sonodynamic therapy (SDT) is a relatively new approach for cancer treatment. It is based on photodynamic therapy, which involves using synergistic effect for killing cancerous cells through a combination of a drug (a sonosensitizer) and ultrasound [3, 4]. Experiments with low- and high-frequency ultrasound waves combined with various sonosensitizers have shown that tumor volume decreases or sonodynamic therapy causes a decline in tumor growth rate [5–7]. A microbubble oscillates during its oscillatory breathing under acoustic pressure. When it reaches the resonant size at ultrasonic frequency, its oscillation amplitude increases to an extreme level followed by its catastrophic collapse, at which the gas inside gets adiabatic compression causing its temperature rise to thousands of degree centigrade. This high temperature leads to the production of free radicals [8] where its surrounding H_2_O molecules start to decompose into °H and °OH, which either recombine, to form HO and H_2,_ or alter the chemistry of drugs [9, 10]. This way the suspended tumor cells are killed by ultrasound at a much higher rate in the presence of porphyrins [11].

The growth of experimental murine tumors was significantly inhibited at low intensity ultrasound waves with a porphyrin dose [12, 13]. Sonoexcitation of the tumor-localizing components of hematoporphyrin (HP) derivative leads to the production of singlet oxygen (^1^O_2_), which is the main agent for induction of necrosis and regression of malignancies [14, 15]. The oxygen effect is important in radiation therapy because malignant tumors usually have a significant population of hypoxic cells. Reoxygenation is a phenomenon in which hypoxic (and thus radioresistant) tumor cells become more exposed to oxygen (and thus more radiosensitive) by coming into closer proximity to capillaries after death of other tumor cells from previous irradiation. If reoxygenation is applied efficiently between dose fractions, the presence of hypoxic cells does not have a significant impact on the outcome of a multifraction regimen [16–18]. Both in vitro and in vivo sonodynamical experiment results have shown that the dual-frequency ultrasound induces more cavitations than the conventional single-frequency ultrasound under the same exposure conditions. According to our previous studies [19], 30-min dual-frequency sonication is a more potent inhibitor of tumor growth than single-frequency sonication with and without Hp injection.

The goal of this study is to assess the therapeutic effect of repeated and fractionated dual-frequency sonication (1 MHz and 150 kHz) at lower level acoustic intensity with hematoporphyrin for the treatment of spontaneous breast adenocarcinoma in Balb/c mice.

## Materials and methods

Hematoporphyrin (Sigma-Aldrich Co., Oakville, Canada) has a purity of 50 % and is dissolved in phosphate buffered saline (PBS, pH = 7.4) and stored in the dark at 4 °C. It was injected at a dose of 10 mg/kg intraperitoneum injection, 6 h before sonication [20].

**Fig. 1:**
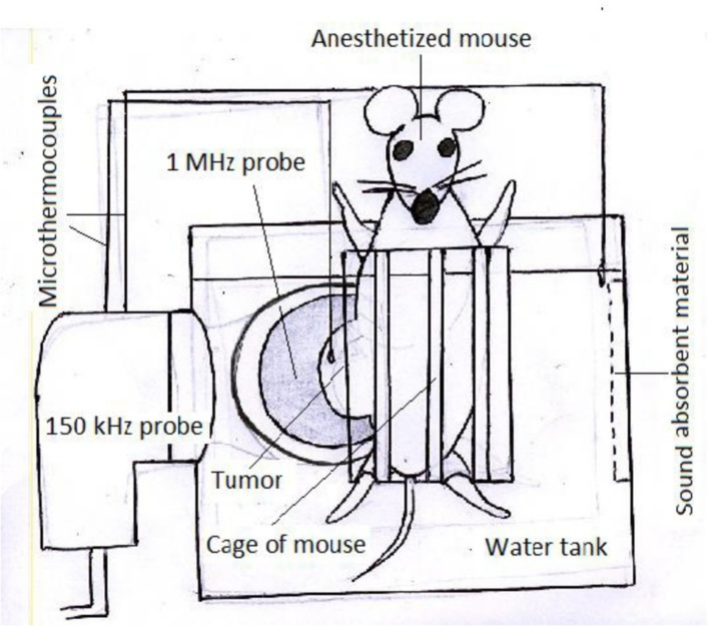
The schematic diagram of the experimental setup

The cubic Perspex water tank (25, 25, and 30 cm^3^) is built in an orthogonal geometry. Two ultrasonic probes are positioned in a way that the central beam axis of each probe is perpendicular to the axis of other. In order to avoid the impact of reflection, the tank walls in front of the probes were covered by a sheet of sound absorbent material (Fig. 1). The first source is a 150 kHz (SM3670, Shrewsbury Medical Ltd., Shropshire, UK), 0.2 W/cm^2^ (0.75 × 10^5^ Pa), and 130 kHz center frequency with a 30-mm diameter and a 5-cm^2^ effective radiation area (ERA). The other source is a 1 MHz (Sonoplus 462 Enrof Nonius Co., Netherlands), 2.0 W/cm^2^ (2.38 × 10^5^ Pa), and a center frequency of 980 kHz with a PZT transducer (30 mm diameter and 5 cm^2^ ERA). Acoustic calibration for the power and intensity of sources was carried out in a degassed water tank using the radiation force balance (UPM-DT-10, Netech, USA, ±1 mW) and the hydrophone method in cubic chamber (Bruel and Kjaer model 8103, Denmark). All reported intensity values consist of the spatial average and temporal average (SATA). For both sources, we were able to change the intensity, mode of sonication, and the duty cycle at the adjusted sonication.

We used repeated and fractionated irradiation with simultaneous dual-frequency ultrasound (continuous mode) for 30-min sonodynamic therapy. In order to keep the temperature of the tumor below the hyperthermia level, the temperature rise during the exposure was checked by thermometer with a 0.1-mm diameter micro-thermocouple (CHY, 502A, Hsin-Chu, Taiwan), which was inserted on the tumor under the skin of the animal. Temperature changes as a result of sonication for 30 min were below the threshold for producing hyperthermia effects (*T* < 35 °C) in all of the experiments.

Breast adenocarcinoma tumor was supplied by the Pasteur Institute, Tehran, Iran. All animal experiments and protocols were evaluated and approved by the Animal and Ethics Review Committee of the Tarbiat Modares University (Tehran, Iran). Each transplanted tumor was initiated with a subcutaneous trocar-injection of an approximately 1–2 cm^3^ piece of fresh tumor into the flank region of female Balb/c inbred mice (6–8 weeks) weighing 19–21 g. When the average volume of tumors reached approximately 1 cm^3^ (*V*0), the tumors were treated. Before treatment, the hair over the tumors was shaved. The tumor-bearing mice were divided into eight groups (72 mice): (A) the control group; (B) the sham group; (C) the injection of HP alone group; (D) 30-min dual-frequency sonication with Hp injection in one repetition at the first day group (US_(150 kHz + 1 MHz)_ + Hp)_0_; (E) two repetitions at the first and sixth days group (US_(150 kHz + 1 MHz)_ + Hp)_0,6_; (F) three repetitions at the first, sixth, and twelfth days group (US_(150 kHz + 1 MHz)_ + Hp)_0,6,12_; (G) four repetitions at the first, sixth, twelfth, and eighteenth days group (US_(150 kHz + 1 MHz)_ + Hp)_0,6,12,18_; and (H) the fractional treatment group treated by 7.5-min dual-frequency sonication and Hp injection at the first, third, sixth, and ninth days group (US_fraction(150 kHz + 1 MHz)_ + Hp)_0,3,6,9_.

Before sonication, mice were anesthetized with an intra-peritoneum (ip) injection of ketamine/xylazine. A tumor-bearing mouse was held vertically in degassed water at 37 °C at 1 cm from each probe [21], so ultrasound waves can propagate through the tumor and the skin without any other acoustic interference.

**Fig. 2:**
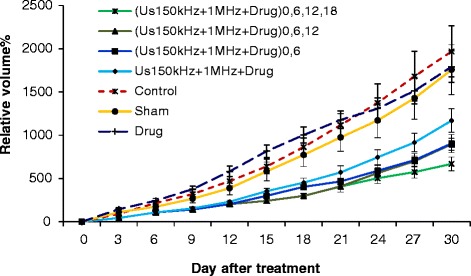
The mean ± SD of the relative volume change of tumor for all groups. (A) The control group; (B) the sham group; (C) the injection of HP alone group; 30-min dual-frequency sonication with Hp injection in (D) one repetition at the first day group (US_(150 kHz + 1 MHz)_ + Hp)_0_; (E) two repetitions at the first and sixth days group (US_(150 kHz + 1 MHz)_ + Hp)_0,6_; (F) three repetitions at the first, sixth, and twelfth days group (US_(150 kHz + 1 MHz)_ + Hp)_0,6,12_; (G) four repetitions at the first, sixth, twelfth, and eighteenth days group (US_(150 kHz + 1 MHz)_ + Hp)_0,6,12,18_

The therapeutic response is evaluated by observing tumor growth for each group after sonodynamic therapy and comparing the result with those from control, sham, and other groups. The length (*a*), width (*b*), and height (*c*) diameters of tumors were measured with a digital caliper every 3 days for 30 days after treatment. The tumor size was calculated by $\mathrm{Volume}=a \times b \times c\frac{\pi }{6}$ and relative volume and inhibition ratio were calculated as follows (Fig. 2):

1 $$ \mathrm{Relative}\ \mathrm{volume}=100\times \frac{V-{V}_0}{V} $$

2 $$ \mathrm{Inhibition}\ \mathrm{ratio}=100\times \frac{{\left(1-\frac{V}{V_0}\right)}_{\mathrm{treatment}\ \mathrm{group}}}{{\left(1-\frac{V}{V_0}\right)}_{\mathrm{sham}\ \mathrm{group}}} $$

${\frac{V}{V_0}}_{\mathrm{treatment}\ \mathrm{group}}$ and ${\frac{V}{V_0}}_{\mathrm{sham}\ \mathrm{group}}$ are normalized tumor volume in the treatment and sham groups, respectively.

To evaluate the delay in tumor growth, the survival time was measured. Survival time was measured from the end of volume calculation time (after 30 days) to death time. For histopathology, tumors were removed from anesthetized mice, fixed in 37 % formaldehyde, and embedded in paraffin wax. Then, sections of tissue were cut and stained with hematoxylin and eosin (H&E). After 30 days, three mice in each group were randomly selected for histopathological examination using the Bloom–Richardson (BR) grading [22] for breast cancer. Tumor grades were determined based on the tumor tubule formation (score 1), number of mitoses/10-high-power fields (score 2), and nuclear grade (score 3). Scores of 3–5, 6–7, and 8–9 indicated well-differentiated (BR low grade), moderately differentiated (BR intermediate grade), and poorly differentiated (BR high grade) tumors, respectively.

Statistical analyses were performed by verifying normality and homogeneity of variables, and one-way analysis of variance (ANOVA) was performed with a 95 % confidence interval. We used survival analysis with the log-rank test for investigating survival times.

## Results

Figure 4 shows the relative volume change in percent versus days after treatment. The results show that the treatment with ultrasound dose repetition of two, three, and four times (E, F, and G) were effective in delaying tumor growth compared with one-time sonication (D) (*p* < 0.05). There were significant differences in the relative volume changes of tumors in these groups compared with the sham, control, and treated with 10 mg/kg intraperitoneum injection of Hp (A, B, and C) (*p* < 0.05) groups. There were significant reductions in growth delay in groups with ultrasound dose repetition from 15th to 30th day after the first treatment (E, F, and G) (*p* < 0.05).

**Fig. 3:**
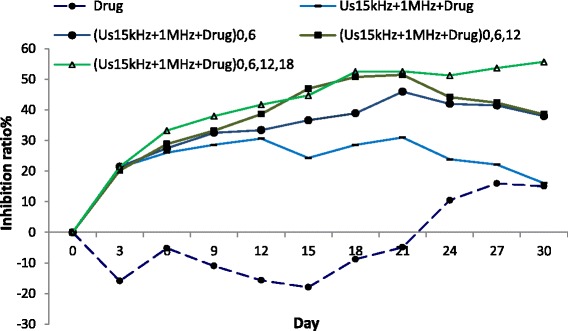
The tumor growth inhibition ratio in the experimental groups. (C) The injection of HP alone group, 30-min dual-frequency sonication with Hp injection in (G) one repetition at the first day group (US_(150 kHz + 1 MHz)_ + Hp)_0_, (E) two repetitions at the first and sixth days group (US_(150 kHz + 1 MHz)_ + Hp)_0,6_, (F) three repetitions at the first, sixth, and twelfth days group (US_(150 kHz + 1 MHz)_ + Hp)_0,6,12_, (G) four repetitions at the first, sixth, twelfth, and eighteenth days group (US_(150 kHz + 1 MHz)_ + Hp)_0,6,12,18_

The results of inhibition ratio in treated groups with repeated dual-frequency sonication with Hp injection were greater than those with Hp injection alone (Fig. 3). In the repeated treatment groups (D, E, F, and G), the inhibition ratio was more than 40 % at day 21 relative to the Hp injection group (C) and reached to 50 % on the 30th day for the four times repeated treatment group. In the treated group with four repetitions (G), the inhibition ratio reached 50 % at the 30th day.

These results demonstrate that repetition of sonodynamic therapy with 30-min dual-frequency sonication and Hp injection is an effective treatment for the control of adenocarcinoma tumor growth relative to other treated groups (C and D).

**Fig. 4:**
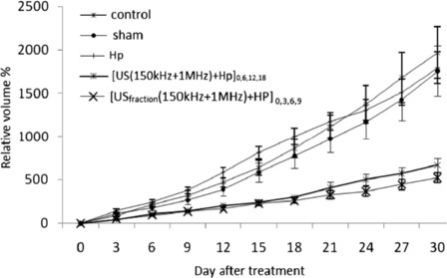
The mean ± SD of the relative volume change of tumor for the five groups. (A) The control group; (B) the sham group; (C) the injection of HP alone group, 30-min dual-frequency sonication with Hp injection in (G) four repetitions at the first, sixth, twelfth, and eighteenth days group (US_(150 kHz + 1 MHz)_ + Hp)_0,6,12,18_; and (H) the fractional treatment group treated by 7.5-min dual-frequency sonication and Hp injection at the first, third, sixth, and ninth days (US_fraction (150 kHz + 1 MHz)_ + Hp)_0,3,6,9_

The treated group with four repetitions (G) was compared with the fractional treatment group (H group) that was treated by 7.5-min dual-frequency sonication and Hp injection at the first, third, sixth, and ninth days (US_fraction (150 kHz + 1 MHz)_ + Hp)_0,3,6,9_ (Fig. 4). The results show that the treatments in the treated groups G and H were effective in delaying tumor growth compared with the control, sham, and Hp injection groups (*p* < 0.05). There was also a significant reduction of the relative volume change of tumor in the ultrasound dose fractionation in group H compared to the ultrasound dose repetition in group G from 24 days after treatment (*p* < 0.05), and there was no significant difference between two groups before this time (*p* > 0.05).

**Fig. 5:**
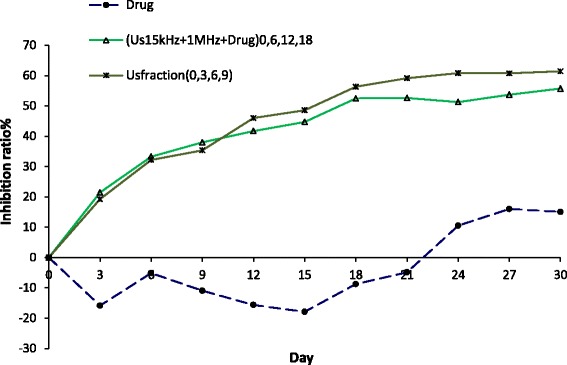
The tumor growth inhibition ratio in the experimental groups. (C) The injection of HP alone group; 30-min dual-frequency sonication with Hp injection in (G) four repetitions at the first, sixth, twelfth, and eighteenth days group (US_(150 kHz + 1 MHz)_ + Hp)_0,6,12,18_; and (H) the fractional treatment group treated by 7.5-min dual-frequency sonication and Hp injection at the first, third, sixth, and ninth days (US_fraction (150 kHz + 1 MHz)_ + Hp)_0,3,6,9_

Figure 5 shows the inhibition ratio versus different days after treatment in experimental groups of injection of Hp alone (Hp), treated with dual-frequency sonication and Hp for four repetitions (30 min/repetition), and four fractions at the first, third, sixth, and ninth days after treatment (7.5 min/fraction). The treated group with fractional therapy (H) had more effective inhibition ratio than the treated group with repetition therapy (G). In other words, the ultrasound dose fractionation is more effective in decreasing tumor growth than the ultrasound dose repetition in four repeats and in one repeat from the 12th to the 30th day. Therefore, the ultrasound dose fractionation regime in sonodynamic therapy could be effective in controlling tumor growth.

**Fig. 6:**
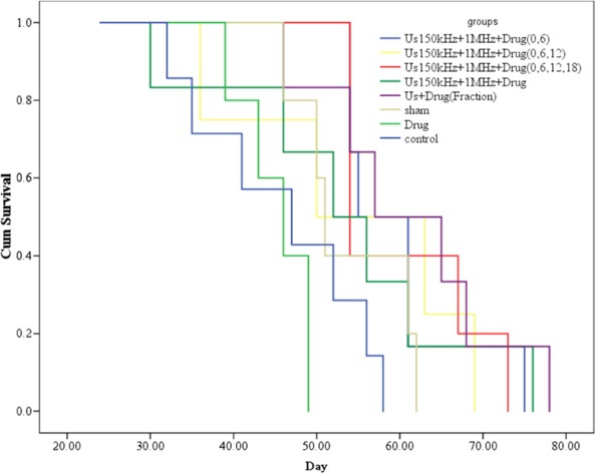
Kaplan–Meier survival analysis of experimental groups. (A) The control group; (B) the sham group; (C) the injection of HP alone group, 30-min dual-frequency sonication with Hp injection in (D) one repetition at the first day group (US_(150 kHz + 1 MHz)_ + Hp)_0_; (E) two repetitions at the first and sixth days group (US_(150 kHz + 1 MHz)_ + Hp)_0,6_; (F) three repetitions at the first, sixth, and twelfth days group (US_(150 kHz + 1 MHz)_ + Hp)_0,6,12_; (G) four repetitions at the first, sixth, twelfth, and eighteenth days group (US_(150 kHz + 1 MHz)_ + Hp)_0,6,12,18_; and (H) the fractional treatment group treated by 7.5-min dual-frequency sonication and Hp injection at the first, third, sixth, and ninth days (US_fraction (150 kHz + 1 MHz)_ + Hp)_0,3,6,9_

Figure 6 shows the results of the Kaplan–Meier survival for all groups. The survival period in the group treated with fractionation dual-frequency sonication with Hp injection was significantly longer than other groups. According to the survival curve, in the period of 50–70 days after the first treatment, 20 % of the mice survived in group E; 75–90 days after the first treatment, 20 % of the mice survived in group F; 50–70 days after the first treatment, 30 % of the mice survived in group G; and 50–80 days after the first treatment, 20 % of the mice survived in group H.

**Fig. 7:**
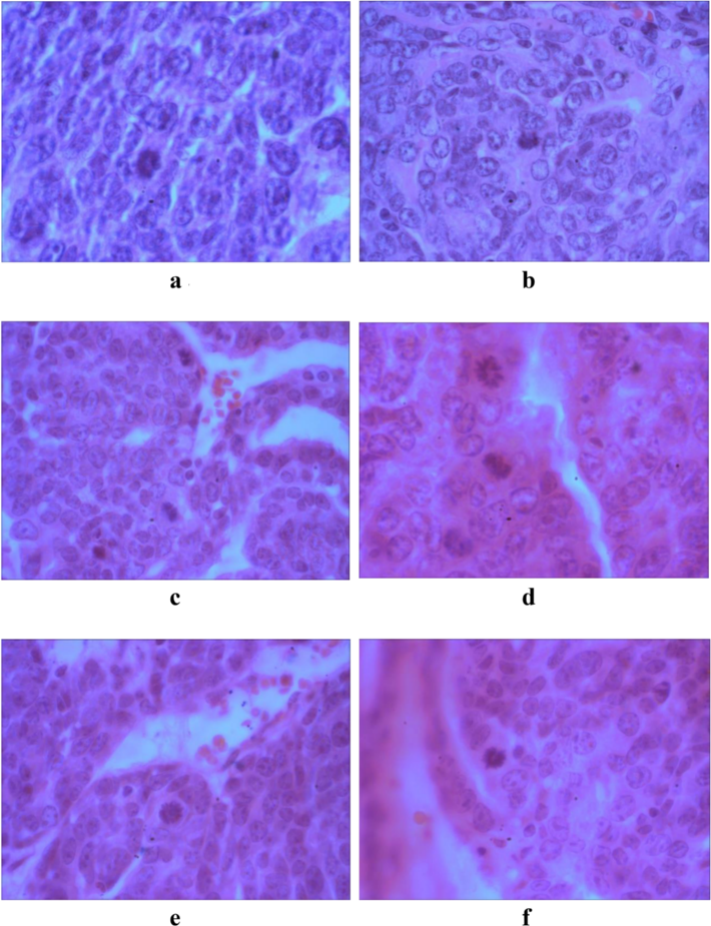
Histopathological images of the tumors on day 30 after treatment from the following experimental groups: **a** the sham group × 200; 30-min dual-frequency sonication with Hp injection in **b** one repetition at the first day group (US_(150 kHz + 1 MHz)_ + Hp)_0_ × 100; **c** two repetitions at the first and sixth days group (US_(150 kHz + 1 MHz)_ + Hp)_0,6_ × 100; **d** three repetitions at the first, sixth, and twelfth days group (US_(150 kHz + 1 MHz)_ + Hp)_0,6,12_ × 400; **e** four repetitions at the first, sixth, twelfth, and eighteenth days group (US_(150 kHz + 1 MHz)_ + Hp)_0,6,12,18_ × 300; and **f** the fractional treatment group treated by 7.5-min dual-frequency sonication and Hp injection at the first, third, sixth, and ninth days (US_fraction (150 kHz + 1 MHz)_ + Hp)_0,3,6,9_ × 400 magnification

To verify sonodynamic therapy results, histopathological studies were performed in the different experimental groups (Fig. 7a–f), based on the Bloom–Richardson classification (Table 1). The sham group (Fig. 7a) showed some nuclear polymorphism. In the group that received Hp injection and dual-frequency sonication (Fig. 7b), the mitotic index of the tumor was reduced. Results for the treated groups with Hp injection and dual-frequency sonication were not very different from groups with two and three repetitions of treatment (Fig. 7c, d). Finally, in the groups that received four repetitions and four fraction Hp with dual-frequency sonication (Fig. 7e, f), although mitotic cells were still present, their nuclear polymorphism was the lowest among all of the groups. All groups were scored based on this classification method including the total tumor grading. Data shows that the treatment with fractionation and four repetitions yields the least score compared with other experimental groups. However, results of pathology indicated that the mitotic activity of tumor cells was reduced following treatment with fractionation and four repetition protocols.

**Table 1:** Bloom–Richardson classification of tumors in sham and treated groups

Group	Tumor tubule formation	Number of mitoses/10-high-power fields	Nuclear grade	Total score	Bloom–Richardson grade	Grade
Sham	2	3	2	7	Moderately differentiated (BR intermediate grade)	2
Dual-frequency sonication + Hp	1	2	3	6	Moderately differentiated (BR intermediate grade)	2
Dual-frequency sonication + Hp (two repetitions)	1	2	3	6	Moderately differentiated (BR intermediate grade)	2
Dual-frequency sonication + Hp (three repetitions)	1	2	3	6	Moderately differentiated (BR intermediate grade)	2
Dual-frequency sonication + Hp (four repetitions)	1	1	2	4	Well differentiated (BR low grade)	1
Dual-frequency sonication + Hp (four fractions)	1	1	2	4	Well differentiated (BR low grade)	1

## Discussion

In recent years, sonodynamic therapy has been widely developed for the treatment of tumors.

In this study, the non-thermal effect of ultrasound based on its inertial cavitation was especially utilized. With ultrasound excitation in the presence of molecular oxygen, the sonosensitizer produces singlet oxygen, which is harmful to the cell function. It is believed that singlet oxygen is the principal cytotoxic species involved in sonodynamic therapy. Previous research has shown that sonodynamic therapy using simultaneous dual- or multi-frequency ultrasound would reduce the growth of tumors [20, 21, 23]. Barati et al. [21] explained that using a low-frequency ultrasound at kHz range induced more transient cavitations than a high-frequency ultrasound. Maximum collapsing of bubbles happens when dual- or multi-frequency ultrasound is used. Barati et al. [24] used sonodynamic therapy on breast adenocarcinoma tumor by using dual-frequency ultrasound and photophyrin sensitizer. Their results showed that using dual-frequency ultrasound is more effective than using single frequency. Also, the relative volume changes of tumors show that in groups with one-time treatment, tumor growth increased at the sixth, twelfth, and eighteenth days after treatment [19]. In this study, these times were selected for repeated sonodynamic therapy when tumors reoxygenated.

In addition, some observations are suggesting that the spaces between fractions are due to reoxygenation of tumors [18]. The mechanisms responsible for this phenomenon have not been clearly established. Suggested mechanisms include reduced oxygen consumption by irradiated cells, rapid cell loss leading to tumor shrinkage, migration of cells from hypoxic to oxygenated areas, and improvements in tumor blood flow [25]. In this study, the antitumor effect of the dual-frequency sonication (1 MHz and 150 kHz) at low-level intensity with the sonosensitizer of Hp was investigated in the mode of dose repetition and fractionation regimes. These effects have not yet been reported in sonodynamic therapy. Accordingly, sonodynamic therapy in four repetitions at the first, sixth, twelfth, and eighteenth days (US_(150 kHz + 1 MHz)_ + Hp)_0,6,12,18_ (30 min/repetition) and four fractions at the first, third, sixth, and ninth days (US_fraction (150 kHz + 1 MHz)_ + Hp)_0,3,6,9_, after treatment (7.5 min/fraction) is more effective than the other exposure regimes (groups C, D, E, and F). The histopathological studies supported our results and showed that the total score of malignancy in the G and H groups was reduced, and cells were more differentiated than those in the other groups.

Indeed, the reduction in tumor growth days after treatment is due to the cell killing because of increase in the level of cavitations which produce free radicals and toxic agents. Our results clearly showed that the repetition and fractionation regimes give tissue the opportunity to repair and resume reoxygenation process for damaged cells caused by sonodynamic therapy. Sonochemical reactions also depend on the presence of oxygen in tumor tissue. However, sonodynamic therapy induces severe hypoxia in tumor tissue after the first irradiation, and this condition is adverse to the induction of a sonochemical reaction. When more days were applied between therapy days, oxygen was induced in cells under hypoxic conditions. Therefore, reoxygenation in hypoxic cells returned to original levels when the oxygen supply was resumed.

## Conclusion

In sonodynamic therapy, the ultrasound dose fractionation and repetition with dual-frequency sonication can have a useful therapeutic effect and may have future clinical applications.

## References

[1] Schattman GL, Navarro J (2008). Breast cancer and fertility preservation. Placenta.

[2] Li C (2010). Breast cancer epidemilogy.

[3] Tachibana KF, Feril LB, Ikeda-Dantsuji Y (2008). Sonodynamic therapy. Ultrasonics.

[4] Rosenthal IS, Sostaric JZ, Riesz P (2004). Sonodynamic therapy: a review of the synergistic effects of drugs and ultrasound. Ultrason Sonochem.

[5] Humphrey VF (2007). Ultrasound and matter: physical interactions. Prog Biophys Mol Biol.

[6] Neppiras EA, Noltingk BE (1951). Cavitation produced by ultrasonics: theoretical conditions for the onset of cavitation. Proc Phys Soc.

[7] Barnett S (1998). Cavitation: its nature, detection and measurement. Ultrasound Med Biol.

[8] Umemura S, Yumita N, Umemura K, Nishigaki R (1999). Sonodynamically induced effect of Rose Bengal on isolated sarcoma 180 cells. Cancer Chemother Pharmacol.

[9] Warthington AE, Thompson J, Lalonde R, Patterson M, Rauth AM, Hunt JW (1997). Mechanism of ultrasound inhanced porphyrin citotoxity, free radical and hematoporphyrin effects. IEEE Ultrason Symp.

[10] Miyoshi N, Tuziuti T, Yasui K, Iida Y, Shimizu N, Riesz P (2008). Ultrasoundinduced cytolysis of cancer cells is enhanced in the presence of micron-sized alumina particles. Ultrason Sonochem.

[11] Yumita N, Nishigaki R, Umemura S (2000). Sonodynamically induced antitumor effect of photofrin II on colon 26 carcinoma. J Cancer Res Clin Oncol.

[12] Tang W, Liu Q, Wang X, Zhang J, Wang P, Mi N (2008). Ultrasound exposure in the presence of hematoporphyrin induced loss of membrane integral proteins and inactivity of cell proliferation associated enzymes in sarcoma 180 cells in vitro. Ultrason Sonochem.

[13] Yumita N, Umemura S, Kaneuchia M, Okanoa Y, Magario N, Shimizua K (1998). Sonodynamically induced cell damage with fluorinated anthracycline derivative FAD104. Cancer Lett.

[14] Lyudmila V, Chekulayeva VAC, Igor N, Shevchuk IN (2008). Active oxygen intermediates in the degradation of hematoporphyrin derivative in tumor cells subjected to photodynamic therapy. J Photochem Photobiol B.

[15] Niedre MS, Patterson AJ, Wilson BC (2005). Singlet oxygen luminescence as an in vivo photodynamic therapy dose metric: Validation in normal mouse skin with topical amino-levulinic acid. Br J Cancer.

[16] Menon C, Fraker DL (2005). Tumor oxygenation status as a prognostic marker. Cancer Lett.

[17] Milus L, Hunter NR, Mason KA, Milross CG, Saito Y, Peters LJ (1995). Role of reoxygenation in induction of enhancement of tumor radioresponse by paclitaxel1. Cancer Res.

[18] Sack G, Thews O, Pöttgen C, Stuschke M, Sack H (1999). Tumour oxygenation during fractionated radiotherapy: comparison with sizematched controls. Acta Oncol.

[19] Alamolhoda M, Mokhtari-Dizaji M, Barati AH, Hasanzadeh H (2012). Comparing the in vivo sonodynamic effects of dual- and single-frequency ultrasound in breast adenocarcinoma. J Med Ultrasound.

[20] Kanthale P, Brotchie B, Ashokkumar M, Grieser F (2008). Experimental and theoretical investigations on sonoluminescence under dual frequency conditions. Ultrason Sonochem.

[21] Barati AH, Mokhtari-Dizaji M, Mozdarani H, Bathaie SZ, Hassan ZM (2007). Effect of exposure parameters on cavitation induced by low-level dual-frequency ultrasound. Ultrason Sonochem.

[22] Bloom-Richardson Grade for Breast Cancer, Cancer Reporting in California (2007). Abstracting and coding procedures for hospitals (California Cancer Reporting System Standards, vol. I).

[23] Zheng H, Mukdadi O, Kim H, Hertzberg JR, Shandas R (2005). Advantages in using multifrequency excitation of contrast microbubbles for enhancing echo particle image velocimetry techniques: initial numerical studies using rectangular and triangular waves. Ultrasound Med Biol.

[24] Barati AH, Mokhtari-Dizaji M (2010). Ultrasound dose fractionation in sonodynamic therapy. Ultrasound Med Bio.

[25] Autieroa M, Celentano L, Cozzolinoc R, Laccettic P, Marottad M, Mettivier G (2007). Early detection of tumor masses by in vivo hematoporphyrin-mediated fluorescence imaging. Nucl Instrum Meth Phys Res A.

